# Adverse drug events associated with tiotropium: a real-world pharmacovigilance study of FDA adverse event reporting system database

**DOI:** 10.3389/jpps.2025.14917

**Published:** 2025-08-29

**Authors:** Yang Rui, Tianyuan Xin, Yu Chen, Beiyi Xiang, Changwen Chen, Lin Zhang, Zhe Chen

**Affiliations:** ^1^ Laboratory of Cough, Kunshan Key Laboratory of Chronic Cough, Affiliated Kunshan Hospital of Jiangsu University, Suzhou, China; ^2^ Department of Oncology, Changshu No.1 People’s Hospital, Changshu Hospital Affiliated to Soochow University, Suzhou, China

**Keywords:** adverse drug events, tiotropium, pharmacovigilance, disproportionality analysis, data mining

## Abstract

**Introduction:**

Tiotropium, a long-acting muscarinic antagonist, is commonly employed for the maintenance treatment of chronic obstructive pulmonary disease (COPD) and asthma. While its efficacy has been validated through numerous randomized controlled trials, safety concerns in real-world post-marketing settings necessitate further evaluation.

**Aim:**

This study aimed to analyze the adverse events (AEs) associated with tiotropium reported in the U.S. Food and Drug Administration Adverse Event Reporting System (FAERS) database to identify potential safety signals.

**Methods:**

A retrospective analysis was conducted on adverse reaction reports related to tiotropium in the FAERS database from the first quarter of 2004 to the fourth quarter of 2024. The AE names in the FAERS database were systematically classified using the Preferred Terms (PTs) and System Organ Classes (SOCs) provided by the latest version of the Medical Dictionary for Regulatory Activities (MedDRA 27.1). After deduplication, a combination of methods, including the Reporting Odds Ratio (ROR), Proportional Reporting Ratio (PRR), Bayesian Confidence Propagation Neural Network (BCPNN), and Multi-item Gamma Poisson Shrinker (MGPS), was employed for disproportionality analysis.

**Results:**

A total of 129,763 AE reports related to tiotropium were included in the analysis, affecting 65,045 patients. These reports encompassed 27 different SOC categories, identifying 264 AEs associated with tiotropium. After excluding certain AEs deemed clinically insignificant, the most common AEs reported were dyspnea (n = 8,600), cough (n = 2,440), and pneumonia (n = 2080). The AEs exhibiting the highest signal strength included aggravated dyspnea (ROR: 162.04), hoarseness (ROR: 43.42), and aggravated chronic obstructive airway disease (ROR: 43.17). Additionally, we identified potential risks not mentioned in the instructions (United States Prescribing Information and the Canadian Product Monograph), such as epiglottic cancer, halo vision, and malignant lung tumors.

**Conclusion:**

This study offers a more comprehensive understanding of tiotropium by uncovering previously unreported adverse reactions. Physicians should take these newly identified adverse reactions into account when prescribing this medication.

## Introduction

Asthma is a significant global public health concern, affecting over 334 million individuals worldwide, as reported in a 2022 study, with an incidence rate of approximately 3.33% [1]. The Global Initiative for Asthma (GINA) recommends tiotropium as an effective add-on therapy for moderate to severe asthma. As a long-acting muscarinic antagonist, tiotropium specifically antagonizes M3 receptors in the airways, thereby inhibiting acetylcholine-induced bronchoconstriction [2, 3]. Compared to short-acting anticholinergic drugs, tiotropium can be effectively combined with β2 receptor agonists to enhance therapeutic efficacy. Previous studies on tiotropium have primarily focused on its indications, resulting in an inadequate description of all adverse events (AEs) associated with this medication [4]. However, reports indicate that some patients have experienced adverse reactions, including dry mouth, constipation, difficulty urinating, and arrhythmia following local inhalation of tiotropium [5–8].

In recent years, pharmacovigilance studies utilizing the U.S. Food and Drug Administration Adverse Event Reporting System (FAERS) have increasingly become a crucial component in the field of international drug safety assessment. The US FAERS is among the largest pharmacovigilance databases worldwide, offering unique advantages in detecting signals of AEs characterized by low incidence, delayed onset, or specific populations [9]. Previous studies leveraging the FAERS database have uncovered significant insights regarding the potential side effects of mepolizumab in the treatment of asthma and eosinophilic granulomatosis with polyangiitis, the safety profile of sulfasalazine in treating autoimmune diseases, and the efficacy of Pralsetinib in real-world scenarios for non-small cell lung cancer and thyroid cancer [10–12]. FAERS studies concerning other drug categories, such as the analysis of deep vein thrombosis associated with JAK inhibitors, have shown that employing multi-method signal detection (combining ROR/PRR/BCPNN) can markedly improve the reliability of rare signal identification [13]. However, most of the common adverse reactions associated with tiotropium are derived from controlled clinical settings involving specific patient populations, where the potential risks of tiotropium may not be fully identified.

## Materials and methods

### Source of data

This study was based on the US FAERS database, utilizing its publicly available quarterly data files from the first quarter of 2004 to the fourth quarter of 2024. We selected the first quarter of 2004 as the starting point in the FAERS database primarily because tiotropium bromide dry powder inhaler (brand name Spiriva Handihaler) was approved by the U.S. FDA during this period as a new chemical entity for the maintenance treatment of COPD [14]. This approval marked tiotropium as the first once-daily long-acting anticholinergic bronchodilator to receive such designation. The keywords “TIOTROPIUM” and “SPIRIVA” were used to extract all AE reports related to tiotropium. The reported data tables include demographic information (DEMO), drug usage records (DRUG), AE records (REAC), patient outcomes (OUTC), duration of drug therapy (THER), drug indications (INDI), and sources of AEs (RPSR) [9]. However, during the data statistical process, records of basic information and AEs that were duplicates or contained issues (as indicated by the quarterly updates from FAERS) were excluded and were not included in the statistical analysis of the aforementioned data tables.

### Data processing

First, the collected reports were sorted by CASEID, FDA_DT, and PRIMARYID. For reports with the same CASEID, the one with the highest FDA_DT value was retained; if both CASEID and FDA_DT were identical, the report with the largest PRIMARYID value was retained [15]. Subsequently, the latest version of the MedDRA dictionary (MedDRA27.1) was applied to encode the AE names in the FAERS database. This primarily involved using the Preferred Terms (PTs) and corresponding SOC from the MedDRA dictionary [16]. Notably, the database had established role_cod (reporting role codes for drugs in incidents) in the DRUG table to identify genuine “drug-adverse event” signals. In this study, we utilized “TIOTROPIUM” and “SPIRIVA” to identify cases in the DRUG file and selected role_cod as the primary suspect drug (PS) to enhance accuracy.

### Statistical analysis

In this study, we analyzed data primarily utilizing the proportional imbalance measure. We employed the Reporting Odds Ratio (ROR), Proportional Reporting Ratio (PRR), Bayesian Confidence Propagation Neural Network (BCPNN), and Multi-item Gamma Poisson Shrinker (MGPS) algorithms to evaluate signals associated with tiotropium-related AEs. These four methods are statistical analysis techniques based on proportional imbalance measurement dichotomous table (Supplementary Table S1), which assess the statistical relationship between a specific drug and a specific AE by calculating the relative frequency of the target adverse reaction caused by the target drug in the database over a defined period. In statistical terms, higher ROR typically indicate a stronger association between drugs and AEs [17]. The PRR determines the incidence of a particular AE for a specific drug at the 95% confidence interval (CI), primarily by analyzing the ratio of the AE rate for exposure to the drug to the rate of AEs that would have occurred without drug exposure [18]. BCPNN assesses the association between a drug and an adverse reaction by calculating the Information Component (IC) [19]. MGPS primarily involves the calculation of the Empirical Bayes Geometric Mean (EBGM), where EBGM05 represents the lower limit of the 95% CI for the EBGM [20]. The specific algorithms of these four methods, along with the criteria for identifying positive signals, are detailed in Supplementary Table S2. In this study, all four conditions must be satisfied simultaneously to confirm the generation of one valid signal. Statistical analyses were conducted using R4.4.2.

## Results

### Baseline characteristic

**FIGURE 1:**
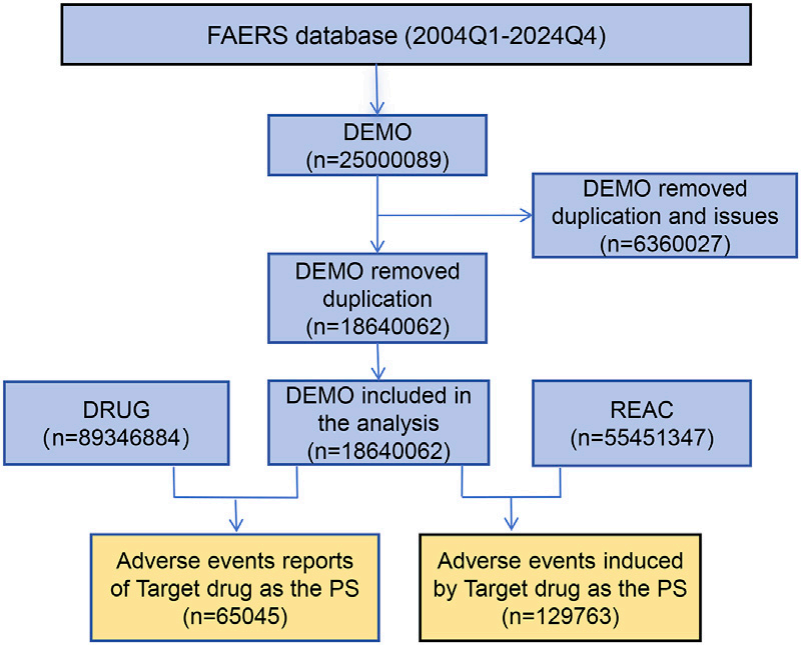
Flowchart of adverse events associated with tiotropium from FAERS. The overall flow of this study in detail. By organizing the data, we ultimately collected 129,763 reports of adverse events associated with tiotropium.

The FAERS database encompasses data from 80 quarters, spanning from the first quarter of 2004 to the fourth quarter of 2024, yielding a total of 25,000,089 items retrieved from the search. After eliminating duplicate entries and addressing problematic data, a total of 18,640,062 complete reports were compiled, which includes 129,763 reports related to tiotropium AEs and 65,045 patient data records. The data cleaning process is depicted in Figure 1.

**FIGURE 2:**
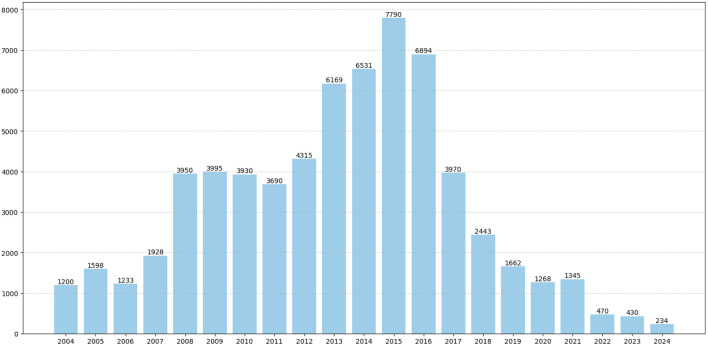
Annual distribution of reported patient cases. The number of reported cases of adverse reactions to tiotropium for each year between the first quarter of 2004 and the fourth quarter of 2024.

Among the collected data from 65,045 patients, the average number of patients reporting AEs annually was 3,097. Notably, between 2013 and 2016, the number of reports increased significantly, with an average annual increase of 6,846 cases (Figure 2). Of the 65,045 cases, females accounted for 59.61%, while males accounted for 34.24%, indicating a higher proportion of females. The age distribution of reporters was primarily concentrated in those aged 45 and above, who accounted for 48.38%. Among the identities of the reporters, consumers constituted the highest proportion, making up 74.12% of the total. We conducted an analysis of the top 5 countries with the highest number of reports. The majority of the reported data originated from the United States, which accounted for 76.01% of the total. Additionally, data reported from Namibia accounted for 14.85% of the total. The onset time interval was primarily concentrated between 30 and 180 days, accounting for 28.46%. In the FAERS database, if a patient has multiple recorded outcomes, the most severe outcome is prioritized for analysis. If a primary outcome appears in different years or quarters, these are considered distinct events. The most common adverse reaction outcomes included hospitalization (12.37%), other medical events (8.37%), and death (3.61%). Specific data are presented in Table 1.

**TABLE 1:** Baseline characteristics of tiotropium-related adverse event (AE) reports based on the FAERS database.

Variables	Amount	Percentage
Sex		
Female	38,771	59.61%
Male	22,274	34.24%
Unknown	4,000	6.15%
Age (years old)		
<18	79	0.12%
18–44	521	0.80%
45–64	8,970	13.79%
65–75	11,294	17.36%
>75	11,204	17.23%
Unknown	32,977	50.70%
Weight (kg)		
<73	6,304	9.69%
73–87	3,339	5.13%
88–104	2039	3.14%
>104	1,436	2.21%
Unknown	51,927	79.83%
Reporter		
Consumer	48,213	74.12%
Pharmacist	6,074	9.34%
Physician	5,791	8.90%
Other health professional	2,484	3.82%
Registered Nurse	4	0.01%
Lawyer	3	0.01%
Unknown	2,476	3.81%
Reporter country(top 5)		
United States of America	49,438	76.01%
Namibia	9,651	14.85%
Canada	1,388	2.13%
Japan	517	0.79%
United Kingdom	263	0.40%
Outcome		
Hospitalization	8,407	12.37%
Other medical events	5,690	8.37%
Death	2,454	3.61%
Life-threatening	436	0.64%
Disability	399	0.59%
Required intervention	30	0.04%
Congenital anomaly	12	0.02%
Unknown	50,559	74.37%

### Signal detection at the SOCs level

We documented AEs associated with tiotropium treatment, encompassing a total of 27 different SOCs (Table 2). Seven SOCs met the criteria of at least one of the four signal detection algorithms. Among these, the categories of injury, poisoning and procedural complications [n = 32,293, ROR: 2.87 (2.83, 2.90), PRR: 2.40 (29,327.90), IC025: 1.24, EBGM05: 2.36], respiratory, thoracic and mediastinal disorders [n = 25,720, ROR: 5.05 (4.98, 5.12), PRR: 4.24 (66,280.14), IC025: 2.05, EBGM05: 4.16], and product issues [n = 6,011, ROR: 2.96 (2.88, 3.03), PRR: 2.87 (7,370.05), IC025: 1.47, EBGM05: 2.78] fulfilled the criteria of the four algorithms. Additionally, infections and infestations, eye disorders, social circumstances, and ear and labyrinth disorders all reached the thresholds set by both algorithms.

**TABLE 2:** Signal strength of tiotropium-related adverse events (AEs) at the System Organ Class (SOC) level in FAERS database.

SOC name	N	ROR (95%CI)	PRR (χ2)	IC025	EBGM05
Injury, poisoning and procedural complications	32,293	2.87 (2.83,2.90)^a^	2.40 (29,327.90)^a^	1.24^a^	2.36^a^
Respiratory, thoracic and mediastinal disorders	25,720	5.05 (4.98,5.12)^a^	4.24 (66,280.14)^a^	2.05^a^	4.16^a^
General disorders and administration site conditions	14,198	0.58 (0.57,0.59)	0.63 (3,814.78)	−0.70	0.62
Gastrointestinal disorders	8,215	0.73 (0.71,0.74)	0.74 (786.70)	−0.46	0.73
Infections and infestations	7,130	1.05 (1.02,1.07)^a^	1.05 (15.45)	0.03^a^	1.02
Product issues	6,011	2.96 (2.88,3.03)^a^	2.87 (7,370.05)^a^	1.47^a^	2.78^a^
Nervous system disorders	5,894	0.51 (0.50,0.53)	0.54 (2,596.61)	−0.94	0.52
Eye disorders	4,599	1.81 (1.76,1.86)^a^	1.78 (1,601.79)	0.79^a^	1.73
Investigations	4,235	0.52 (0.50,0.53)	0.53 (1863.13)	−0.96	0.52
Musculoskeletal and connective tissue disorders	3,006	0.43 (0.42,0.45)	0.45 (2,166.40)	−1.21	0.43
Psychiatric disorders	2,924	0.39 (0.37,0.40)	0.40 (2,784.68)	−1.37	0.39
Cardiac disorders	2,897	0.85 (0.82,0.88)	0.85 (78.84)	−0.29	0.82
Skin and subcutaneous tissue disorders	2,346	0.32 (0.31,0.34)	0.33 (3,282.50)	−1.64	0.32
Neoplasms benign, malignant and unspecified (incl cysts and polyps)	2,271	0.66 (0.63,0.69)	0.67 (385.53)	−0.64	0.64
Renal and urinary disorders	2006	0.81 (0.77,0.85)	0.81 (88.95)	−0.36	0.78
Vascular disorders	1,207	0.43 (0.41,0.46)	0.44 (899.62)	−1.28	0.41
Metabolism and nutrition disorders	1,172	0.41 (0.39,0.43)	0.42 (981.44)	−1.35	0.39
Social circumstances	700	1.16 (1.08,1.25)^a^	1.16 (15.03)	0.10^a^	1.07
Ear and labyrinth disorders	669	1.19 (1.10,1.28)^a^	1.19 (20.26)	0.14^a^	1.10
Surgical and medical procedures	592	0.33 (0.31,0.36)	0.34 (781.37)	−1.68	0.31
Immune system disorders	552	0.38 (0.35,0.42)	0.39 (547.03)	−1.50	0.35
Reproductive system and breast disorders	438	0.38 (0.34,0.42)	0.38 (446.65)	−1.53	0.35
Blood and lymphatic system disorders	295	0.13 (0.12,0.15)	0.13 (1,673.60)	−3.06	0.12
Hepatobiliary disorders	175	0.15 (0.13,0.17)	0.15 (877.32)	−2.98	0.13
Endocrine disorders	147	0.44 (0.38,0.52)	0.44 (102.32)	−1.40	0.38
Congenital, familial and genetic disorders	56	0.14 (0.11,0.19)	0.14 (288.82)	−3.17	0.11
Pregnancy, puerperium and perinatal conditions	15	0.03 (0.02,0.04)	0.03 (529.83)	−5.84	0.02

^a^ indicates statistically significant signals in algorithm; FAERS, FDA, adverse event reporting system; N, the number of reports; ROR, reporting odds ratio; PRR, proportional reporting ratio; 95% CI, 95% confidence interval; χ2, chi-squared statistic; IC025, the lower limit of the 95% confidence interval for the IC; EBGM05, the lower limit of the 95% confidence interval for the EBGM.

### Signal detection at the PTs level

Through comprehensive analysis of the database, we identified a total of 264 AEs associated with tiotropium, all of which met the criteria established by the four algorithms (For details, refer to Supplementary Table S3). Notably, this includes some rare AEs such as vascular perforation, oral mucosal roughness, bronchial malformation, and congenital hiatal hernia. We examined the distribution of the top 30 PTs based on the frequency of AEs linked to tiotropium (Table 3). After excluding PTs unrelated to non-drug treatments, the ten AEs with the highest signal frequencies were dyspnoea (n = 8,600), cough (n = 2,440), pneumonia (n = 2080), chronic obstructive pulmonary disease (n = 1909), dry mouth (n = 1893), asthma (n = 1,338), wheezing (n = 1,121), dysphonia (n = 1,048), blurred vision (n = 847), and chest discomfort (n = 704). Furthermore, we ranked the AE signals by intensity, with the top 30 signals based on EBGM05 presented in Table 4. After excluding PTs related to non-drug treatments, the top ten AE signals exhibiting the highest intensity included exacerbated dyspnoea (EBGM: 96.93), hoarseness (EBGM: 29.06), exacerbated chronic obstructive airways disease (EBGM: 27.46), abnormal FEV1/FVC ratio (EBGM: 20.51), airway burns (EBGM: 20.45), halo vision (EBGM: 19.52), increased viscosity of nasal secretions (EBGM: 18.38), increased upper airway secretions (EBGM: 18.41), epiglottic carcinoma (EBGM: 16.56), and chronic obstructive pulmonary disease (EBGM: 16.56).

**TABLE 3:** Signal strength of tiotropium-related adverse events (AEs) at the Preferred Term (PT) level in the FAERS database, ranked by frequency for the top 30 events.

PT name	N	ROR (95%CI)	PRR (χ2)	IC025	EBGM05
incorrect route of drug administration	23,258	965.01 (943.5,987.02)	792.23 (6,434,342.04)	8.08	271.63
dyspnoea	8,600	7.79 (7.62,7.96)	7.34 (46,701.42)	2.82	7.07
product quality issue	4,923	17.85 (17.34,18.38)	17.21 (72,433.37)	4.0	16.11
cough	2,440	4.32 (4.15,4.49)	4.25 (6,041.0)	2.02	4.06
pneumonia	2080	3.16 (3.03,3.3)	3.13 (3,002.2)	1.57	2.98
chronic obstructive pulmonary disease	1909	18.29 (17.46,19.15)	18.03 (29,493.39)	4.04	16.56
dry mouth	1893	11.69 (11.16,12.24)	11.53 (17,748.9)	3.42	10.75
asthma	1,338	6.29 (5.96,6.64)	6.24 (5,807.35)	2.54	5.84
wheezing	1,121	9.86 (9.29,10.47)	9.79 (8,652.34)	3.16	9.04
dysphonia	1,048	8.79 (8.26,9.34)	8.72 (7,028.44)	3.0	8.06
incorrect route of product administration	875	29.57 (27.6,31.67)	29.38 (22,442.67)	4.64	25.72
vision blurred	847	3.0 (2.81,3.21)	2.99 (1,115.63)	1.47	2.78
chest discomfort	704	3.36 (3.12,3.62)	3.35 (1,151.78)	1.62	3.09
lung neoplasm malignant	671	7.58 (7.03,8.19)	7.55 (3,749.5)	2.77	6.89
product physical issue	670	17.49 (16.18,18.89)	17.4 (9,953.51)	3.92	15.51
bronchitis	643	4.07 (3.76,4.4)	4.05 (1,466.72)	1.89	3.72
oropharyngeal pain	597	3.1 (2.86,3.36)	3.09 (836.66)	1.49	2.83
productive cough	572	6.11 (5.62,6.63)	6.08 (2,397.56)	2.45	5.54
cataract	566	4.72 (4.34,5.13)	4.7 (1,633.14)	2.09	4.29
therapeutic product effect incomplete	561	4.06 (3.74,4.42)	4.05 (1,277.72)	1.88	3.7
urinary retention	497	7.26 (6.64,7.93)	7.23 (2,626.3)	2.69	6.52
loss of personal independence in daily activities	421	4.69 (4.26,5.16)	4.68 (1,205.1)	2.06	4.21
throat irritation	390	4.27 (3.87,4.72)	4.26 (965.28)	1.92	3.83
dysuria	386	4.97 (4.49,5.49)	4.96 (1,205.28)	2.13	4.44
dyspnoea exertional	384	5.01 (4.53,5.54)	5.0 (1,215.67)	2.15	4.48
blood count abnormal	329	9.57 (8.58,10.68)	9.55 (2,463.3)	3.03	8.39
eye pain	320	2.93 (2.62,3.27)	2.92 (402.47)	1.37	2.61
sleep disorder due to a general medical condition	303	12.83 (11.44,14.38)	12.8 (3,200.23)	3.42	11.11
obstructive airways disorder	300	13.82 (12.32,15.51)	13.79 (3,447.73)	3.52	11.93
lung disorder	297	2.95 (2.63,3.31)	2.95 (379.74)	1.38	2.62

FAERS, FDA, adverse event reporting system; N, the number of reports; ROR, reporting odds ratio; PRR, proportional reporting ratio; 95% CI, 95% confidence interval; χ2, chi-squared statistic; IC025, the lower limit of the 95% confidence interval for the IC; EBGM05, the lower limit of the 95% confidence interval for the EBGM.

**TABLE 4:** Signal strength of tiotropium-related adverse events (AEs) at the Preferred Term (PT) level in the FAERS database, ranked by EBGM05 for the top 30 events.

PT name	N	ROR (95%CI)	PRR (χ2)	IC025	EBGM05
incorrect route of drug administration	23,258	965.01 (943.5,987.02)	792.23 (6,434,342.04)	8.08	271.63
dyspnoea exacerbated	142	162.04 (133.57,196.59)	161.87 (16,454.78)	5.74	96.93
capsule issue	12	124.79 (65.58,237.45)	124.78 (1,139.83)	2.64	50.85
capsule physical issue	22	76.27 (48.45,120.06)	76.25 (1,385.98)	3.45	41.19
hoarseness	45	43.42 (31.95,59.01)	43.4 (1,691.99)	3.98	29.06
chronic obstructive airways disease exacerbated	33	43.17 (30.17,61.75)	43.16 (1,234.0)	3.69	27.46
incorrect route of product administration	875	29.57 (27.6,31.67)	29.38 (22,442.67)	4.64	25.72
product quality control issue	17	42.39 (25.75,69.78)	42.38 (624.81)	2.93	23.47
fev1/fvc ratio abnormal	4	68.21 (23.74,196.01)	68.21 (228.37)	0.83	20.51
airway burns	5	59.21 (23.24,150.9)	59.21 (251.25)	1.19	20.45
halo vision	61	26.82 (20.71,34.74)	26.81 (1,426.05)	3.8	19.52
increased viscosity of nasal secretion	6	47.37 (20.38,110.11)	47.37 (245.11)	1.46	18.38
increased upper airway secretion	242	22.02 (19.35,25.06)	21.98 (4,609.88)	4.09	18.41
epiglottic carcinoma	3	63.95 (19.0,215.21)	63.95 (161.65)	0.36	16.56
chronic obstructive pulmonary disease	1909	18.29 (17.46,19.15)	18.03 (29,493.39)	4.04	16.56
product quality issue	4,923	17.85 (17.34,18.38)	17.21 (72,433.37)	4.0	16.11
total lung capacity abnormal	17	27.99 (17.13,45.72)	27.98 (415.07)	2.75	16.11
residual urine	9	33.66 (17.08,66.35)	33.66 (264.32)	2.01	15.86
intraocular pressure test	13	29.02 (16.55,50.9)	29.02 (329.26)	2.44	15.53
lung carcinoma cell type unspecified recurrent	29	24.06 (16.55,34.97)	24.05 (606.54)	3.18	15.7
reversible airways obstruction	24	24.96 (16.54,37.67)	24.96 (521.38)	3.04	15.66
product physical issue	670	17.49 (16.18,18.89)	17.4 (9,953.51)	3.92	15.51
lung hyperinflation	37	20.65 (14.85,28.73)	20.65 (659.78)	3.24	14.19
small airways disease	5	34.95 (14.04,86.97)	34.94 (152.38)	1.15	13.01
abnormal loss of weight	212	15.79 (13.77,18.11)	15.77 (2,827.42)	3.63	13.29
eosinophil count	6	31.2 (13.62,71.47)	31.19 (163.4)	1.4	12.72
eosinophil count abnormal	28	19.8 (13.55,28.92)	19.8 (477.54)	3.0	12.98
total lung capacity increased	10	25.53 (13.49,48.33)	25.53 (222.36)	2.06	12.75
sputum retention	33	17.86 (12.61,25.3)	17.85 (503.93)	3.03	12.12
obstructive airways disorder	300	13.82 (12.32,15.51)	13.79 (3,447.73)	3.52	11.93

FAERS, FDA adverse event reporting system; N, the number of reports; ROR, reporting odds ratio; PRR, proportional reporting ratio; 95% CI, 95% confidence interval; χ2, chi-squared statistic; IC025, the lower limit of the 95% confidence interval for the IC; EBGM05, the lower limit of the 95% confidence interval for the EBGM.

### Event occurrence time

**FIGURE 3:**
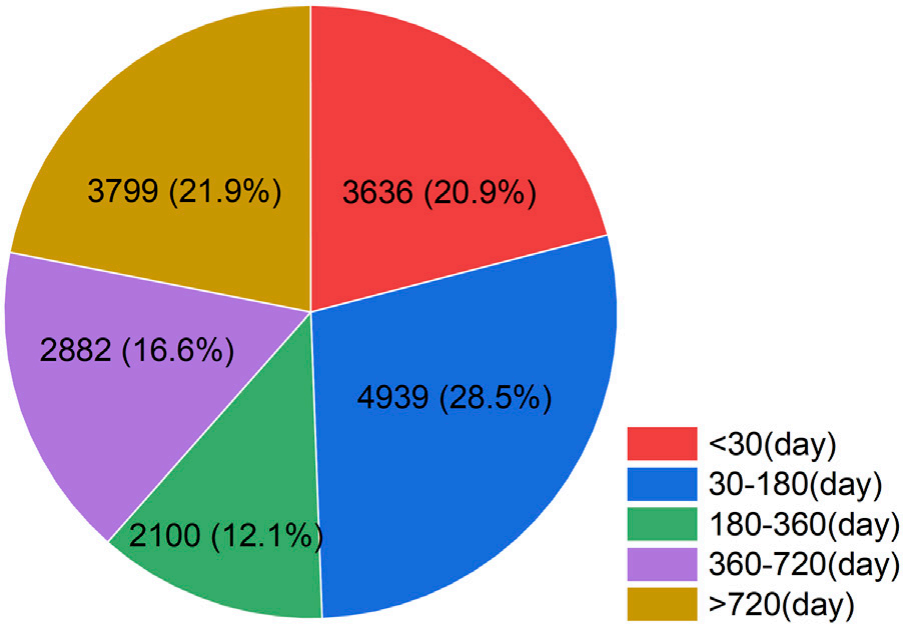
Time to event occurrence (day). The temporal distribution of adverse events associated with tiotropium. Herein, the numbers represent the count of AE reports and their corresponding percentages during the specified period, after excluding reports with incomplete data.

Regarding the temporal distribution of AEs associated with Tiotropium, we collected a total of 17,356 reports after excluding those with incomplete data (Figure 3). Among these reports, 28.5% (n = 4,939) of the AEs occurred within 1–6 months after initiating tiotropium treatment. Additionally, 21.9% (n = 3,799) of the AEs were still observed after 2 years of tiotropium treatment.

## Discussion

This study systematically analyzed the safety signals of tiotropium using the FAERS database, revealing the complex risk profile of tiotropium in real-world settings. We compared the tiotropium instructions in this study with official reference documents, including the United States Prescribing Information (USPI)^1^ and the Canadian Product Monograph (PM)^2^. Our findings indicated that AEs such as dry mouth, upper respiratory tract infections, sinusitis, glaucoma, urinary retention, and bronchospasm were consistent with the results of this study. We also observed that AEs explicitly mentioned in both labeling, such as tachycardia, gastroesophageal reflux, urticaria, anaphylactic shock, and constipation, did not occur in this study. Additionally, we identified potential risks not mentioned in either labeling, including epiglottic cancer, halo vision, and malignant lung tumors. Furthermore, in interpreting the data results, we excluded PTs that were unrelated to non-pharmacological treatments, such as “incorrect route of administration.” These PTs do not represent direct medical errors; rather, they are issues that can be effectively addressed by providing users with more precise guidance or information.

A large-scale real-world study based on the FAERS revealed a significant gender distribution difference in tiotropium-related AE reports: among the 65,045 included reports, females accounted for 59.61%, significantly higher than males at 34.24%. This finding is noteworthy as it contradicts the descriptions provided in drug labeling documents, such as the USPI and the PM, which do not emphasize gender differences. Meanwhile, the PM, based on pharmacokinetic summary analysis, demonstrated no gender difference in the systemic exposure to tiotropium. In response to this contradiction, we speculate that it may be related to differences in reporting behavior. Female patients are more inclined to proactively report subjective symptoms (such as dry mouth and blurred vision) and non-fatal AEs, whereas male patients may underestimate or overlook such discomforts. We also observed a significant increase in the number of tiotropium-related AE reports from 2013 to 2016, with a peak of 7,790 cases in 2015. This trend may be associated with the findings of a 2013 study on the tiotropium Respimat inhaler and its risk of death in patients with COPD. The study confirmed no significant difference in mortality risk between the Respimat doses of 5 μg or 2.5 μg and the HandiHaler 18 μg [21]. This discovery may have heightened public vigilance towards the drug. Additionally, the FDA’s approval of tiotropium for asthma patients aged 12 years and older in 2015 significantly expanded the population eligible for its use [22]. Consumers are the primary source of AE reports, which facilitates the direct observation of patients' intuitive feedback in real-world settings. The majority of AE reports originate from the United States, accounting for 76.01% of the total. This predominance can be primarily attributed to the FAERS system, which serves as the safety reporting database in the U.S. The domestic population’s heightened awareness and utilization of the FAERS system likely contribute to this elevated reporting rate, which falls within the anticipated range. The majority of AEs associated with tiotropium primarily occur during the initial phase of medication use; therefore, enhanced monitoring is required during this phase to promptly identify potential AEs, such as worsening dyspnea. The highest proportion of affected patients is among those aged 65 and above, which may be related to the decline in renal function in the older adults population that could affect drug clearance rates. In terms of outcomes, hospitalization is relatively common. Older adults patients often have concomitant cardiovascular, cerebrovascular, and pulmonary diseases, leading to a significant increase in hospitalization rates.

We identified a significant association signal between increased difficulty in breathing and tiotropium (ROR: 162.04, EBGM05: 96.93). It is noteworthy that the underlying mechanisms of this association remain unclear. The hypotheses presented herein are intended solely to elucidate potential pathways rather than to draw definitive conclusions. Tiotropium, an anticholinergic drug, exerts sustained inhibition of M3 receptors, potentially altering the activity of macrophages and other inflammatory cells. This alteration may lead to an increased release of pro-inflammatory cytokines, such as IL-33 and TNF-α, thereby exacerbating inflammation and airway hyperresponsiveness [23]. Excessive inhibition of M3 receptors can also reduce Cl-secretion, affecting the viscoelasticity of mucus [24]. Prolonged use of anticholinergic drugs may foster a type 2 inflammatory environment, induce structural changes in the airways, and result in excessive mucus secretion [25]. A clinical study indicated that approximately 36% of patients exhibited airway mucus plugs on CT scans [26]. Additionally, the antagonistic effect of tiotropium on M3 receptors in airway smooth muscle may enhance vagal tone through a negative feedback mechanism, potentially leading to paradoxical bronchoconstriction in patients [27, 28]. The activation of M2 receptors inhibits acetylcholine release, inducing negative feedback regulation of smooth muscle contraction. However, tiotropium exhibits a slight antagonistic effect on the M2 receptor, which may disrupt this negative feedback regulation, leading to an increase in acetylcholine release from nerve terminals and consequently enhancing smooth muscle contraction via M3 receptor activation [29, 30]. Moreover, tiotropium may increase heart rate by blocking M2 receptors, thereby elevating cardiac workload and myocardial oxygen consumption [31]. This effect could exacerbate the oxygen demand in patients with cardiopulmonary diseases, ultimately triggering symptoms of dyspnea. Therefore, for patients with combined cardiopulmonary diseases who have received initial treatment with tiotropium, it is recommended to assess dyspnea 24 h post-treatment, and the combined use of mucolytic agents should be considered if necessary.

We also identified several AEs that, although not included in the tiotropium product label, exhibited significant signal strengths, such as epiglottis cancer (ROR: 63.95, EBGM05: 16.56) and malignant lung tumors (ROR: 7.58, EBGM05: 6.89). However, there are currently no relevant animal models or *in vitro* experiments available to validate this association. Consequently, we propose the following plausible speculations regarding this strong signal association. Long-term use of tiotropium can lead to a reduction in intracellular calcium levels by inhibiting the Gq/11 signaling pathway [32]. This reduction in calcium ion concentration inhibits the activity of Src family kinases (SFKs), resulting in the abnormal activation of transcription factor 3 (STAT3) through altered JAK-STAT3 binding and Janus-activated kinase (JAK) activity [33–35]. Activated STAT3 can translocate to the nucleus, bind to the promoter regions of target genes, and regulate the expression of transcription factors such as SNAI and TWIST [36]. These transcription factors can inhibit the expression of epithelial cell markers (such as E-cadherin and Claudin) while upregulating mesenchymal cell markers (such as Vimentin and N-cadherin), thereby promoting the transition of cells from an epithelial phenotype to a mesenchymal phenotype [37]. Furthermore, M3 receptors are also present in immune cells, such as dendritic cells (DCs), which possess antigen-presenting functions [38]. Tiotropium reduces the ability of DCs to activate T cells by inhibiting the M3 receptor and decreasing the expression of MHC-II molecules (such as HLA-DR) and co-stimulatory molecules (such as CD80/CD86) [39, 40]. The interaction between the NF-κB and STAT3 signaling pathways can increase the expression of immunosuppressive molecules (such as PD-L1), thereby inhibiting the activation and proliferation of T cells [41]. Following the activation of the M3 receptor, it can also regulate the migration of DCs to the draining lymph nodes by upregulating the expression of chemokine receptors, such as CCR7 [42]. When Tiotropium blocks this pathway, DCs are retained in peripheral tissues, resulting in impaired immune surveillance. For patients on long-term medication, future considerations should include whole-genome sequencing and methylation analysis of bronchoalveolar lavage fluid to capture early mutation signals (such as EGFR and KRAS). Whole genome sequencing enables the simultaneous screening of multiple lung cancer driver genes, such as KRAS G12C, with 88% of these genes exhibiting a detection sensitivity exceeding 99% [43]. Consequently, compared to traditional imaging examinations, whole genome sequencing can facilitate the earlier identification of precancerous lesions. For instance, the early detection of EGFR mutations can prompt the initiation of targeted therapy with third-generation tyrosine kinase inhibitors, such as Osimertinib, thereby significantly improving the survival rate of lung cancer patients [44]. Furthermore, long-term exposure to anticholinergic drugs may induce hypermethylation in the promoter region of tumor suppressor genes, such as RASSF1A, in airway epithelial cells [45]. Methylation analysis of bronchoalveolar lavage fluid can assist in the diagnosis of lung cancer. For example, when the methylation of SHOX2 and RASSF1A is combined for lung cancer diagnosis, the sensitivity reaches 81.0%, and the specificity is 97.4%, significantly outperforming the serum biomarker carcinoembryonic antigen (CEA) with a sensitivity of 30.6% and a specificity of 100.0% [46].

In conclusion, based on the real-world risk evidence presented in this study, it is recommended that tiotropium manufacturers collaborate with the FDA, patients, and healthcare providers moving forward. Pharmaceutical enterprises should submit algorithm-validated high-risk signals to the FDA on a quarterly basis, accompanied by relevant case details and epidemiological context. Physicians are encouraged to improve the reporting mechanisms for AEs in clinical practice, which should include the implementation of electronic medical record pop-up reminders for AEs. Patients must be vigilant about any discomfort symptoms following medication intake, communicate promptly with their physicians, and ensure proper reporting of related AEs. In the meantime, the FDA needs to streamline the AE reporting system to better align the upload interface and operational procedures with public needs. Furthermore, the FDA is required to incorporate a “Real-World Evidence” section into drug labeling based on the relevant AEs submitted by pharmaceutical companies, physicians, and individuals, utilizing a three-color system to grade the credibility of risks.

### Limitations

This study uncovered the real-world safety risks associated with tiotropium by analyzing the FAERS database; however, it is not without limitations. First, FAERS, as a spontaneous reporting system, relies heavily on reports from healthcare professionals or patients, which can result in incomplete or biased data collection. Second, the majority of reports originated from the United States, while relatively few were reported from other countries, potentially leading to statistical bias. Regarding the global applicability of the study findings, further validation could be achieved by integrating multiple databases, including the JADER database in Japan and the Canada Vigilance database in Canada. Additionally, although four statistical methods were employed in this study to mitigate bias, these tests can only reveal associations and cannot establish causality. The mechanisms concerning the potential induction of specific AEs, such as dyspnea and a rare type of cancer, by tiotropium are merely speculative hypotheses rather than established causal relationships. These mechanistic hypotheses may provide a foundation for future research investigations. Future research should consider integrating prospective clinical trials and studies on biological mechanisms to validate the causality of high signaling drugs.

## Conclusion

We conducted a comprehensive analysis of AEs associated with tiotropium using the FAERS database. Our findings are generally consistent with the AEs listed in the drug insert. In the clinical application of tiotropium, physicians should closely monitor common AEs, including worsening dyspnea, hoarseness, and exacerbation of chronic obstructive pulmonary disease. Additionally, we identified several previously unreported AEs, such as malignant lung tumors, epiglottic cancer, visual vertigo, and macular degeneration. In clinical practice, clinicians should carefully assess risks when prescribing medications, particularly for high-risk populations. Although the results offer important perspectives for clinical application, additional confirmation via extensive prospective studies is required in the future.

## Data Availability

The original contributions presented in the study are included in the article/Supplementary Material, further inquiries can be directed to the corresponding authors.

## Footnotes

1
https://www.accessdata.fda.gov/drugsatfda_docs/label/2025/021395Orig1s078lbl.pdf

2
https://pdf.hres.ca/dpd_pm/00076710.PDF
