# Supplementary material for: Adverse drug events associated with tiotropium: a real-world pharmacovigilance study of FDA adverse event reporting system database
Source: J Pharm Pharm Sci. 2025 Aug 29;28:14917. doi: 10.3389/jpps.2025.14917 (PMC12425831; doi:10.3389/jpps.2025.14917)
Supplement: Supplementary file 1 [file Table1.docx]

**Supplementary Table S1. Proportional Imbalance Measurement Dichotomous Table**

| Type of drug | targeted adverse event | other adverse events | Total |
| --- | --- | --- | --- |
| Target drug | a | b | a+b |
| Other drugs | c | d | c+d |
| Total | a+c | b+d | a+b+c+d |

a, number of reports containing both the suspect drug and the suspect adverse drug reaction; b, number of reports containing the suspect adverse drug reaction with other medications (except the drug of interest); c, number of reports containing the suspect drug with other adverse drug reactions (except the event of interest); d, number of reports containing other medications and other adverse drug reactions.
