# Supplementary material for: Adverse drug events associated with tiotropium: a real-world pharmacovigilance study of FDA adverse event reporting system database
Source: J Pharm Pharm Sci. 2025 Aug 29;28:14917. doi: 10.3389/jpps.2025.14917 (PMC12425831; doi:10.3389/jpps.2025.14917)
Supplement: Supplementary file 2 [file Table2.docx]

**Supplementary Table S2. Four statistical algorithms and signal generation conditions**

| Methods | Calculation formula | Algorithmic signal generation conditions |
| --- | --- | --- |
| ROR | $ROR=\frac{ad}{bc}$ $SE(\ln ROR)=\sqrt{(\frac{1}{a}+\frac{1}{b}+\frac{1}{c}+\frac{1}{d})}$ $95\%CI=e^{\ln(ROR)\pm\sqrt[1.96]{(\frac{1}{a}+\frac{1}{b}+\frac{1}{c}+\frac{1}{d})}}$     | 95%CI (lower limit)>1、  a≥3 |
| PRR | $PRR=\frac{a/(a+b)}{c/(c+d)}$   | a≥3、PRR>2 、χ 2≥4 |
| BCPNN | $\begin{matrix} IC=\log_{2}\frac{a(a+b+c+d)}{(a+c)(a+b)} \\ E(IC)=\log_{2}\frac{(a+\gamma11)(a+b+c+d+\alpha)(a+b+c+d+\beta)}{(a+b+c+d+\gamma)(a+b+\alpha1)(a+c+\beta1)} \\ V(IC)=\frac{1}{(\ln2)^{2}}\{[\frac{(a+b+c+d)-a+\gamma+\gamma11}{(a+\gamma11)(1+a+b+c+d+\gamma)}]+[\frac{(a+b+c+d)-(a+b)+\alpha-\alpha1}{(a+b+\alpha1)(1+a+b+c+d+\alpha)}]+[\frac{(a+b+c+d)-(a+c)+\beta-\beta1}{(a+c+\beta1)(1+a+b+c+d+\beta)}]\} \end{matrix}$ | IC-2SD (IC025) >0 |
| MGPS | $\begin{matrix} EBGM=a(a+b+c+d)/(a+c)/(a+b) \\ 95\%CI=e^{\ln(EBGM)\pm\sqrt[1.96]{(\frac{1}{a}+\frac{1}{b}+\frac{1}{c}+\frac{1}{d})}} \end{matrix}$ | EBGM05>2 |

ROR,reporting odds ratio;PRR,proportional reporting ratio;BCPNN,bayesian confidence propagation neural network;MGPS,multi-item gamma poisson shrinker;CI,confidence interval ;IC,information component.
