# Supplementary material for: Adverse drug events associated with tiotropium: a real-world pharmacovigilance study of FDA adverse event reporting system database
Source: J Pharm Pharm Sci. 2025 Aug 29;28:14917. doi: 10.3389/jpps.2025.14917 (PMC12425831; doi:10.3389/jpps.2025.14917)
Supplement: Supplementary file 3 [file Table3.docx]

**Supplementary Table S3. Summary of All Signals for 264 Types of AEs**

| PT name | N | ROR | PRR | IC025 | EBGM05 |
| --- | --- | --- | --- | --- | --- |
| incorrect route of drug administration | 23258 | 965.01 | 792.23 | 8.08 | 271.63 |
| dyspnoea | 8600 | 7.79 | 7.34 | 2.82 | 7.07 |
| product quality issue | 4923 | 17.85 | 17.21 | 4.00 | 16.11 |
| cough | 2440 | 4.32 | 4.25 | 2.02 | 4.06 |
| pneumonia | 2080 | 3.16 | 3.13 | 1.57 | 2.98 |
| chronic obstructive pulmonary disease | 1909 | 18.29 | 18.03 | 4.04 | 16.56 |
| dry mouth | 1893 | 11.69 | 11.53 | 3.42 | 10.75 |
| asthma | 1338 | 6.29 | 6.24 | 2.54 | 5.84 |
| wheezing | 1121 | 9.86 | 9.79 | 3.16 | 9.04 |
| dysphonia | 1048 | 8.79 | 8.72 | 3.00 | 8.06 |
| incorrect route of product administration | 875 | 29.57 | 29.38 | 4.64 | 25.72 |
| vision blurred | 847 | 3.00 | 2.99 | 1.47 | 2.78 |
| chest discomfort | 704 | 3.36 | 3.35 | 1.62 | 3.09 |
| lung neoplasm malignant | 671 | 7.58 | 7.55 | 2.77 | 6.89 |
| product physical issue | 670 | 17.49 | 17.40 | 3.92 | 15.51 |
| bronchitis | 643 | 4.07 | 4.05 | 1.89 | 3.72 |
| oropharyngeal pain | 597 | 3.10 | 3.09 | 1.49 | 2.83 |
| productive cough | 572 | 6.11 | 6.08 | 2.45 | 5.54 |
| cataract | 566 | 4.72 | 4.70 | 2.09 | 4.29 |
| therapeutic product effect incomplete | 561 | 4.06 | 4.05 | 1.88 | 3.70 |
| urinary retention | 497 | 7.26 | 7.23 | 2.69 | 6.52 |
| loss of personal independence in daily activities | 421 | 4.69 | 4.68 | 2.06 | 4.21 |
| throat irritation | 390 | 4.27 | 4.26 | 1.92 | 3.83 |
| dysuria | 386 | 4.97 | 4.96 | 2.13 | 4.44 |
| dyspnoea exertional | 384 | 5.01 | 5.00 | 2.15 | 4.48 |
| blood count abnormal | 329 | 9.57 | 9.55 | 3.03 | 8.39 |
| eye pain | 320 | 2.93 | 2.92 | 1.37 | 2.61 |
| sleep disorder due to a general medical condition | 303 | 12.83 | 12.80 | 3.42 | 11.11 |
| obstructive airways disorder | 300 | 13.82 | 13.79 | 3.52 | 11.93 |
| lung disorder | 297 | 2.95 | 2.95 | 1.38 | 2.62 |
| oxygen saturation decreased | 286 | 2.54 | 2.53 | 1.16 | 2.25 |
| medication error | 274 | 2.38 | 2.37 | 1.06 | 2.10 |
| ageusia | 273 | 5.21 | 5.20 | 2.17 | 4.57 |
| extra dose administered | 270 | 3.78 | 3.78 | 1.72 | 3.33 |
| macular degeneration | 257 | 11.05 | 11.03 | 3.19 | 9.52 |
| dry throat | 251 | 12.15 | 12.13 | 3.32 | 10.43 |
| hypoacusis | 248 | 2.81 | 2.81 | 1.29 | 2.47 |
| increased upper airway secretion | 242 | 22.02 | 21.98 | 4.09 | 18.41 |
| aphonia | 239 | 8.43 | 8.42 | 2.82 | 7.28 |
| glaucoma | 227 | 5.66 | 5.66 | 2.26 | 4.91 |
| swollen tongue | 222 | 3.36 | 3.35 | 1.53 | 2.92 |
| visual acuity reduced | 219 | 2.95 | 2.94 | 1.34 | 2.56 |
| pulmonary congestion | 216 | 7.75 | 7.74 | 2.69 | 6.66 |
| abnormal loss of weight | 212 | 15.79 | 15.77 | 3.63 | 13.29 |
| choking | 212 | 5.27 | 5.27 | 2.15 | 4.55 |
| lung infection | 206 | 5.72 | 5.72 | 2.26 | 4.93 |
| emphysema | 202 | 9.40 | 9.39 | 2.94 | 8.01 |
| glossodynia | 187 | 4.52 | 4.52 | 1.93 | 3.88 |
| haemoptysis | 172 | 2.90 | 2.90 | 1.29 | 2.48 |
| pulmonary mass | 166 | 5.47 | 5.47 | 2.17 | 4.64 |
| laryngitis | 156 | 7.12 | 7.11 | 2.52 | 5.98 |
| dyspnoea exacerbated | 142 | 162.04 | 161.87 | 5.74 | 96.93 |
| dementia | 135 | 2.40 | 2.40 | 1.00 | 2.02 |
| sputum discoloured | 134 | 7.02 | 7.01 | 2.48 | 5.83 |
| oral pain | 131 | 2.73 | 2.73 | 1.17 | 2.29 |
| pharyngeal oedema | 129 | 3.64 | 3.64 | 1.57 | 3.04 |
| pharyngolaryngeal pain | 129 | 10.46 | 10.46 | 3.00 | 8.59 |
| bronchospasm | 124 | 4.06 | 4.06 | 1.72 | 3.38 |
| feeling jittery | 116 | 2.82 | 2.82 | 1.20 | 2.34 |
| pneumothorax | 115 | 3.49 | 3.48 | 1.49 | 2.88 |
| oral candidiasis | 108 | 4.12 | 4.12 | 1.71 | 3.38 |
| candida infection | 103 | 3.12 | 3.12 | 1.32 | 2.56 |
| tongue disorder | 90 | 5.68 | 5.68 | 2.11 | 4.56 |
| tongue discolouration | 89 | 9.34 | 9.34 | 2.76 | 7.42 |
| oral discomfort | 88 | 2.98 | 2.98 | 1.23 | 2.40 |
| prostatomegaly | 86 | 10.55 | 10.54 | 2.91 | 8.33 |
| secretion discharge | 85 | 3.54 | 3.54 | 1.46 | 2.84 |
| candidiasis | 78 | 8.28 | 8.28 | 2.57 | 6.51 |
| prostatic specific antigen increased | 76 | 2.57 | 2.57 | 1.00 | 2.04 |
| nasal dryness | 74 | 5.69 | 5.69 | 2.07 | 4.47 |
| intraocular pressure increased | 74 | 2.70 | 2.69 | 1.06 | 2.14 |
| eosinophil count increased | 73 | 4.27 | 4.27 | 1.68 | 3.36 |
| upper-airway cough syndrome | 66 | 4.04 | 4.03 | 1.58 | 3.14 |
| pulmonary function test decreased | 66 | 7.16 | 7.16 | 2.34 | 5.53 |
| benign prostatic hyperplasia | 65 | 5.97 | 5.97 | 2.10 | 4.62 |
| respiration abnormal | 65 | 4.75 | 4.75 | 1.80 | 3.69 |
| hearing impaired | 64 | 4.58 | 4.58 | 1.75 | 3.55 |
| aortic aneurysm | 61 | 3.68 | 3.68 | 1.44 | 2.84 |
| halo vision | 61 | 26.82 | 26.81 | 3.80 | 19.52 |
| sputum increased | 60 | 14.97 | 14.97 | 3.19 | 11.20 |
| bronchiectasis | 59 | 4.68 | 4.68 | 1.76 | 3.59 |
| urine flow decreased | 59 | 11.82 | 11.81 | 2.92 | 8.90 |
| dementia alzheimer's type | 54 | 2.74 | 2.74 | 1.01 | 2.09 |
| visual disturbance | 54 | 4.01 | 4.01 | 1.53 | 3.05 |
| glossitis | 49 | 6.55 | 6.55 | 2.13 | 4.88 |
| sensation of foreign body | 48 | 2.80 | 2.80 | 1.02 | 2.10 |
| foreign body | 48 | 5.23 | 5.23 | 1.84 | 3.90 |
| hoarseness | 45 | 43.42 | 43.40 | 3.98 | 29.06 |
| total lung capacity decreased | 42 | 13.23 | 13.22 | 2.89 | 9.46 |
| accidental exposure | 42 | 4.34 | 4.34 | 1.56 | 3.18 |
| oedema mouth | 39 | 5.21 | 5.21 | 1.77 | 3.76 |
| carotid artery occlusion | 39 | 4.98 | 4.98 | 1.71 | 3.60 |
| breath sounds abnormal | 39 | 3.93 | 3.93 | 1.40 | 2.85 |
| stomach discomfort | 38 | 3.14 | 3.13 | 1.10 | 2.27 |
| lung hyperinflation | 37 | 20.65 | 20.65 | 3.24 | 14.19 |
| prostatic disorder | 36 | 2.98 | 2.98 | 1.02 | 2.14 |
| forced expiratory volume decreased | 35 | 6.31 | 6.31 | 1.96 | 4.47 |
| choking sensation | 34 | 2.97 | 2.97 | 0.99 | 2.11 |
| sputum retention | 33 | 17.86 | 17.85 | 3.03 | 12.12 |
| vocal cord disorder | 33 | 7.87 | 7.87 | 2.19 | 5.49 |
| lung neoplasm | 33 | 3.48 | 3.47 | 1.19 | 2.45 |
| chronic obstructive airways disease exacerbated | 33 | 43.17 | 43.16 | 3.69 | 27.46 |
| oxygen saturation abnormal | 32 | 6.01 | 6.00 | 1.86 | 4.19 |
| small cell lung cancer | 32 | 11.86 | 11.85 | 2.62 | 8.13 |
| bronchial secretion retention | 32 | 13.31 | 13.31 | 2.74 | 9.10 |
| nasal polyps | 31 | 4.51 | 4.51 | 1.50 | 3.14 |
| bronchial obstruction | 31 | 10.31 | 10.31 | 2.46 | 7.07 |
| pulmonary function test abnormal | 31 | 10.00 | 10.00 | 2.42 | 6.86 |
| viral upper respiratory tract infection | 31 | 2.97 | 2.97 | 0.97 | 2.08 |
| oral fungal infection | 30 | 5.14 | 5.14 | 1.65 | 3.55 |
| urinary tract disorder | 30 | 3.26 | 3.26 | 1.07 | 2.26 |
| increased bronchial secretion | 29 | 5.28 | 5.28 | 1.67 | 3.62 |
| lung carcinoma cell type unspecified recurrent | 29 | 24.06 | 24.05 | 3.18 | 15.70 |
| throat cancer | 28 | 3.01 | 3.01 | 0.95 | 2.07 |
| bronchitis chronic | 28 | 5.41 | 5.41 | 1.68 | 3.69 |
| eosinophil count abnormal | 28 | 19.80 | 19.80 | 3.00 | 12.98 |
| foreign body aspiration | 27 | 17.99 | 17.99 | 2.89 | 11.77 |
| tongue coated | 27 | 7.90 | 7.89 | 2.09 | 5.31 |
| blood immunoglobulin e increased | 26 | 5.34 | 5.34 | 1.63 | 3.59 |
| heart valve incompetence | 25 | 4.12 | 4.12 | 1.30 | 2.76 |
| oesophageal disorder | 25 | 3.04 | 3.04 | 0.92 | 2.04 |
| hypopnoea | 25 | 3.54 | 3.54 | 1.11 | 2.38 |
| lung carcinoma cell type unspecified stage iv | 24 | 3.23 | 3.23 | 0.98 | 2.15 |
| carbon dioxide increased | 24 | 8.68 | 8.68 | 2.13 | 5.69 |
| reversible airways obstruction | 24 | 24.96 | 24.96 | 3.04 | 15.66 |
| chronic sinusitis | 23 | 3.09 | 3.09 | 0.91 | 2.04 |
| pharyngeal erythema | 23 | 4.76 | 4.76 | 1.44 | 3.13 |
| capsule physical issue | 22 | 76.27 | 76.25 | 3.45 | 41.19 |
| suffocation feeling | 21 | 4.00 | 4.00 | 1.19 | 2.58 |
| respiratory tract irritation | 21 | 10.02 | 10.01 | 2.18 | 6.36 |
| urinary tract obstruction | 21 | 4.21 | 4.21 | 1.25 | 2.72 |
| hypercapnia | 20 | 3.20 | 3.20 | 0.90 | 2.05 |
| restrictive pulmonary disease | 20 | 8.38 | 8.38 | 1.98 | 5.29 |
| therapeutic reaction time decreased | 20 | 3.72 | 3.72 | 1.08 | 2.38 |
| tongue blistering | 20 | 4.13 | 4.13 | 1.20 | 2.64 |
| angle closure glaucoma | 20 | 3.71 | 3.71 | 1.07 | 2.37 |
| urinary hesitation | 20 | 3.83 | 3.83 | 1.11 | 2.45 |
| foreign body in respiratory tract | 20 | 6.72 | 6.72 | 1.75 | 4.26 |
| nocturnal dyspnoea | 19 | 7.11 | 7.11 | 1.78 | 4.46 |
| respiratory symptom | 19 | 3.72 | 3.72 | 1.06 | 2.36 |
| mycobacterium avium complex infection | 19 | 3.63 | 3.63 | 1.02 | 2.30 |
| painful respiration | 18 | 3.42 | 3.42 | 0.93 | 2.14 |
| breath odour | 17 | 3.30 | 3.30 | 0.86 | 2.03 |
| open angle glaucoma | 17 | 12.99 | 12.99 | 2.25 | 7.80 |
| increased viscosity of bronchial secretion | 17 | 13.81 | 13.80 | 2.30 | 8.27 |
| oxygen consumption decreased | 17 | 13.04 | 13.04 | 2.25 | 7.82 |
| product quality control issue | 17 | 42.39 | 42.38 | 2.93 | 23.47 |
| tongue dry | 17 | 6.00 | 5.99 | 1.53 | 3.67 |
| total lung capacity abnormal | 17 | 27.99 | 27.98 | 2.75 | 16.11 |
| bronchial wall thickening | 16 | 11.52 | 11.52 | 2.10 | 6.84 |
| bronchopulmonary aspergillosis allergic | 16 | 10.35 | 10.35 | 2.01 | 6.17 |
| traumatic lung injury | 16 | 3.90 | 3.90 | 1.02 | 2.37 |
| laryngeal cancer | 16 | 6.57 | 6.57 | 1.59 | 3.96 |
| vital capacity decreased | 15 | 16.96 | 16.96 | 2.32 | 9.76 |
| sputum purulent | 14 | 10.17 | 10.17 | 1.88 | 5.86 |
| aortic valve disease | 14 | 4.00 | 4.00 | 0.98 | 2.35 |
| collapse of lung | 14 | 7.25 | 7.25 | 1.59 | 4.21 |
| eosinophilic granulomatosis with polyangiitis | 13 | 3.62 | 3.62 | 0.83 | 2.08 |
| oropharyngeal blistering | 13 | 3.53 | 3.53 | 0.80 | 2.04 |
| oropharyngeal swelling | 13 | 5.87 | 5.87 | 1.33 | 3.36 |
| tobacco user | 13 | 3.83 | 3.83 | 0.89 | 2.20 |
| bronchial hyperreactivity | 13 | 5.02 | 5.02 | 1.18 | 2.88 |
| intraocular pressure test | 13 | 29.02 | 29.02 | 2.44 | 15.53 |
| chronic respiratory failure | 12 | 5.10 | 5.10 | 1.14 | 2.86 |
| sputum abnormal | 12 | 8.70 | 8.70 | 1.62 | 4.83 |
| asthma-chronic obstructive pulmonary disease overlap syndrome | 12 | 14.62 | 14.62 | 2.00 | 7.97 |
| product blister packaging issue | 12 | 5.72 | 5.72 | 1.25 | 3.20 |
| foreign body trauma | 12 | 7.06 | 7.06 | 1.45 | 3.93 |
| capsule issue | 12 | 124.79 | 124.78 | 2.64 | 50.85 |
| neovascular age-related macular degeneration | 11 | 4.28 | 4.27 | 0.90 | 2.34 |
| bladder discomfort | 11 | 4.31 | 4.31 | 0.91 | 2.36 |
| tracheostomy | 11 | 3.72 | 3.72 | 0.76 | 2.04 |
| dust allergy | 11 | 6.89 | 6.89 | 1.35 | 3.74 |
| lip exfoliation | 11 | 3.98 | 3.98 | 0.83 | 2.18 |
| leukoplakia oral | 11 | 11.14 | 11.14 | 1.73 | 5.98 |
| bladder prolapse | 11 | 3.86 | 3.86 | 0.80 | 2.12 |
| lung diffusion test decreased | 10 | 13.93 | 13.93 | 1.77 | 7.20 |
| total lung capacity increased | 10 | 25.53 | 25.53 | 2.06 | 12.75 |
| bronchospasm paradoxical | 10 | 16.72 | 16.72 | 1.87 | 8.57 |
| respiratory gas exchange disorder | 10 | 12.73 | 12.73 | 1.72 | 6.60 |
| mycotic allergy | 9 | 6.21 | 6.21 | 1.10 | 3.18 |
| dyspnoea paroxysmal nocturnal | 9 | 5.56 | 5.56 | 1.00 | 2.85 |
| vocal cord thickening | 9 | 14.43 | 14.42 | 1.67 | 7.20 |
| residual urine | 9 | 33.66 | 33.66 | 2.01 | 15.86 |
| squamous cell carcinoma of lung | 9 | 5.59 | 5.59 | 1.01 | 2.87 |
| forced vital capacity decreased | 8 | 6.35 | 6.35 | 1.01 | 3.12 |
| micturition frequency decreased | 8 | 4.92 | 4.92 | 0.80 | 2.43 |
| diaphragmatic disorder | 8 | 4.87 | 4.87 | 0.79 | 2.41 |
| bronchial irritation | 8 | 18.74 | 18.74 | 1.65 | 8.86 |
| throat lesion | 8 | 5.41 | 5.41 | 0.88 | 2.66 |
| spirometry abnormal | 8 | 8.66 | 8.66 | 1.24 | 4.22 |
| mite allergy | 8 | 8.08 | 8.08 | 1.19 | 3.95 |
| small cell lung cancer stage unspecified | 8 | 9.34 | 9.34 | 1.29 | 4.55 |
| vocal cord dysfunction | 8 | 7.48 | 7.48 | 1.14 | 3.66 |
| irregular breathing | 8 | 7.03 | 7.03 | 1.09 | 3.45 |
| oxygen saturation | 8 | 14.33 | 14.33 | 1.53 | 6.87 |
| eye redness | 7 | 11.01 | 11.01 | 1.24 | 5.08 |
| keratoconjunctivitis sicca | 7 | 8.20 | 8.20 | 1.07 | 3.82 |
| chronic obstructive airways disease | 7 | 10.74 | 10.73 | 1.23 | 4.96 |
| postnasal drip | 7 | 5.48 | 5.48 | 0.77 | 2.57 |
| nasal mucosal disorder | 7 | 6.04 | 6.04 | 0.85 | 2.83 |
| croup infectious | 7 | 4.57 | 4.57 | 0.62 | 2.15 |
| asbestosis | 7 | 12.59 | 12.59 | 1.31 | 5.78 |
| prostatic adenoma | 7 | 19.38 | 19.38 | 1.49 | 8.71 |
| mucosal discolouration | 7 | 5.15 | 5.15 | 0.73 | 2.42 |
| blood cholesterol | 6 | 6.21 | 6.21 | 0.72 | 2.74 |
| pneumonectomy | 6 | 15.89 | 15.89 | 1.22 | 6.80 |
| forced expiratory volume abnormal | 6 | 20.63 | 20.63 | 1.30 | 8.69 |
| eosinophil count | 6 | 31.20 | 31.19 | 1.40 | 12.72 |
| eosinophilic pneumonia chronic | 6 | 10.19 | 10.19 | 1.02 | 4.44 |
| mouth breathing | 6 | 6.77 | 6.77 | 0.78 | 2.98 |
| peak expiratory flow rate decreased | 6 | 6.15 | 6.15 | 0.72 | 2.71 |
| lower respiratory tract infection bacterial | 6 | 5.50 | 5.50 | 0.64 | 2.43 |
| self-induced vomiting | 6 | 13.53 | 13.53 | 1.15 | 5.83 |
| increased viscosity of nasal secretion | 6 | 47.37 | 47.37 | 1.46 | 18.38 |
| device interaction | 5 | 9.65 | 9.65 | 0.78 | 3.90 |
| bullous lung disease | 5 | 17.33 | 17.33 | 1.00 | 6.83 |
| corneal dystrophy | 5 | 5.58 | 5.58 | 0.47 | 2.28 |
| bladder neck obstruction | 5 | 19.03 | 19.03 | 1.03 | 7.46 |
| breath sounds decreased | 5 | 23.69 | 23.68 | 1.08 | 9.14 |
| biopsy lung | 5 | 6.29 | 6.29 | 0.54 | 2.57 |
| lung lobectomy | 5 | 6.64 | 6.64 | 0.58 | 2.71 |
| prostate examination abnormal | 5 | 8.92 | 8.92 | 0.74 | 3.61 |
| respiratory tract inflammation | 5 | 5.19 | 5.19 | 0.42 | 2.13 |
| small airways disease | 5 | 34.95 | 34.94 | 1.15 | 13.01 |
| benign lung neoplasm | 5 | 8.96 | 8.96 | 0.74 | 3.63 |
| airway burns | 5 | 59.21 | 59.21 | 1.19 | 20.45 |
| eosinophilic bronchitis | 5 | 12.61 | 12.61 | 0.89 | 5.05 |
| lung diffusion disorder | 4 | 13.75 | 13.75 | 0.63 | 4.93 |
| neurosensory hypoacusis | 4 | 16.24 | 16.24 | 0.67 | 5.78 |
| carbon monoxide diffusing capacity decreased | 4 | 10.34 | 10.34 | 0.53 | 3.75 |
| peripheral arthritis | 4 | 19.16 | 19.16 | 0.71 | 6.75 |
| fev1/fvc ratio abnormal | 4 | 68.21 | 68.21 | 0.83 | 20.51 |
| carbon dioxide abnormal | 4 | 5.96 | 5.96 | 0.27 | 2.20 |
| presbyacusis | 4 | 6.99 | 6.99 | 0.36 | 2.57 |
| prolonged expiration | 4 | 7.41 | 7.41 | 0.39 | 2.72 |
| bladder distension | 4 | 14.09 | 14.09 | 0.63 | 5.05 |
| oxygen consumption | 4 | 11.60 | 11.60 | 0.57 | 4.19 |
| inspiratory capacity decreased | 4 | 8.44 | 8.44 | 0.45 | 3.08 |
| expiratory reserve volume decreased | 4 | 33.44 | 33.44 | 0.80 | 11.23 |
| tongue black hairy | 4 | 14.96 | 14.96 | 0.65 | 5.34 |
| vessel perforation | 4 | 13.75 | 13.75 | 0.63 | 4.93 |
| vocal cord inflammation | 4 | 6.53 | 6.53 | 0.32 | 2.40 |
| vocal cord polyp | 4 | 6.56 | 6.56 | 0.32 | 2.41 |
| pulmonary function test | 3 | 31.20 | 31.19 | 0.35 | 9.02 |
| pulmonary function test increased | 3 | 35.53 | 35.53 | 0.36 | 10.12 |
| mediastinum neoplasm | 3 | 8.70 | 8.70 | 0.10 | 2.72 |
| congenital hiatus hernia | 3 | 14.06 | 14.05 | 0.24 | 4.32 |
| blood immunoglobulin e decreased | 3 | 10.15 | 10.15 | 0.15 | 3.16 |
| decreased bronchial secretion | 3 | 39.97 | 39.97 | 0.36 | 11.22 |
| mononeuritis | 3 | 8.76 | 8.76 | 0.10 | 2.74 |
| sight disability | 3 | 7.94 | 7.94 | 0.07 | 2.49 |
| buccal mucosal roughening | 3 | 9.20 | 9.20 | 0.12 | 2.88 |
| lung hernia | 3 | 26.65 | 26.65 | 0.34 | 7.83 |
| sputum decreased | 3 | 35.53 | 35.53 | 0.36 | 10.12 |
| bronchomalacia | 3 | 10.66 | 10.66 | 0.17 | 3.31 |
| exposure via inhalation | 3 | 6.84 | 6.84 | 0.01 | 2.16 |
| airway remodelling | 3 | 13.19 | 13.19 | 0.22 | 4.06 |
| laryngeal obstruction | 3 | 8.36 | 8.36 | 0.09 | 2.62 |
| necrotising ulcerative gingivostomatitis | 3 | 13.46 | 13.46 | 0.23 | 4.14 |
| upper respiratory fungal infection | 3 | 7.75 | 7.75 | 0.06 | 2.44 |
| breath sounds | 3 | 10.40 | 10.40 | 0.16 | 3.24 |
| epiglottic carcinoma | 3 | 63.95 | 63.95 | 0.36 | 16.56 |
| cerebral congestion | 3 | 10.48 | 10.48 | 0.16 | 3.26 |
